# Perioperative management for elderly patients undergoing day surgery: evidence-based practice for nursing care and day surgery

**DOI:** 10.3389/fmed.2023.1298778

**Published:** 2023-11-21

**Authors:** Mei Wu, Lu Xia, Li Zhang, Yamin Xu, Yun Cheng, Xiaoju Zhang, Long Chen

**Affiliations:** ^1^Day Surgery Unit, Huadong Hospital Affiliated to Fudan University, Shanghai, China; ^2^Day Chemotherapy Unit, Huadong Hospital Affiliated to Fudan University, Shanghai, China; ^3^School of Medicine, The Chinese University of Hong Kong, Shenzhen, China; ^4^Department of Nursing, Fudan University Shanghai Cancer Center, Shanghai, China; ^5^Department of Oncology, Shanghai Medical College, Fudan University, Shanghai, China; ^6^Medical Affairs Department, Huadong Hospital Affiliated to Fudan University, Shanghai, China

**Keywords:** evidence, practice, elderly, day surgery, patients, care, nursing, management

## Abstract

**Introduction:**

It is necessary to explore the evidence-based practice of perioperative management of elderly patients undergoing ambulatory surgery, to optimize the clinical workflow and improve the quality of nursing care.

**Methods:**

Based on the best evidence obtained from the previous study, the perioperative management model and examination index of elderly patients undergoing day surgery were established, and the evidence of best practice was obtained by using the evidence-based methodology. Then, we integrated evidence into clinical practice and improved the process. We made a comparative analysis of the effect before and after the implementation of the evidence.

**Results:**

This study summarized 26 pieces of evidence of perioperative management of elderly patients undergoing day surgery and transformed the evidence into 7 items and 11 items of examination index. After the application of the best evidence, knowledge, belief, and practice of perioperative management of nurses for elderly patients in the day operation ward reached 100%, and the rate of reaching the standard of most indicators increased after the application of evidence. The length of waiting for admission and waiting for operation and returning to the ward to discharge of elderly patients decreased significantly, and the difference was statistically significant (All *p* < 0.05).

**Conclusion:**

Evidence-based perioperative management of elderly patients undergoing day surgery improves the nursing practice of clinical nurses and shortens the waiting time and hospitalization time of elderly patients undergoing day surgery, which should be promoted in clinical nursing care.

## Introduction

In recent years, with the shortage of medical resources and the rise of medical expenses, how to reduce medical expenses, speed up patient turnover, and make full and rational use of bed resources has become an important issue that the public is concerned about and the government urgently needs to solve (1, 2). The promotion of the day surgery ward has become one of the effective ways to solve this contradiction. The China Ambulatory Surgery Alliance (CASA) defines day surgery as “an operation or operation completed by a patient entering or leaving the hospital in 1 day (24 h)” (3). The longest hospitalization time of patients who need to be delayed due to their illness is not more than 48 h. At present, China is vigorously promoting the development of day surgery. From 2015 to 2017, China Government has issued many policy documents to promote the development of day surgery and expand the scope of day surgery (4). With the development of day surgery, it is very important to have standardized medical and nursing management processes to ensure the care quality and safety of patients (5). From the preparation of pre-admission examination and operation scheduling to the follow-up of hospitalization and discharge on the day of operation, nurses are important participants in all aspects that directly affect the smooth implementation and development of daytime surgery (6, 7). It is necessary to standardize management and care processes in order to ensure the quality of medical care and promote the development of high efficiency and quality of day surgery (8, 9).

Under the trend of the aging population and development of medical technology, more and more elderly patients can tolerate surgical treatment. For the elderly, shorter hospital stay can reduce the incidence of postoperative complications and readmission rates (10). At present, a large number of medical institutions have actively explored and practiced day surgery, but the perioperative management of day surgery in elderly patients is more complex because of their pathophysiological particularity (11). Compared with young patients, elderly patients need more medical management services (12). Therefore, the purpose of this study is to apply the best evidence of perioperative management of elderly patients undergoing day surgery through a systematic transformation of clinical evidence, to optimize the clinical workflow to improve the operational efficiency of the day operation ward and improve the quality of nursing care for elderly patients undergoing day surgery.

## Methods

This study obtained approval from the ethical committee of our hospital with approval number: HD190026. This study was a before and after self-control study design. All the procedures were performed in accordance with the related regulations and guidelines.

### Evidence acquisition

#### Identify the problem

We defined evidence-based problems and retrieval strategies according to the PIPOST principles. P (population): the target of evidence application was elderly patients undergoing day surgery; I (intervention): intervention, evidence-based practice based on the best evidence, including perioperative workflow management before admission, during hospital stay, and after discharge; P (professional): the implementer of clinical transformation of evidence, nurse, doctor, and anesthesiologist; O (outcome): including waiting time for surgery, preparation for discharge, patient satisfaction, practitioners’ knowledge, and belief and evidence-based compliance; S (setting): clinical scenario and day surgery ward of our hospital; T (type): including clinical practice guide, expert consensus, evidence summary, and systematic evaluation.

#### Search for evidence

According to the “6s” evidence model, evidence retrieval was carried out. The English search strategies were (“old” OR “elderly” OR “aged” OR “geriatric”) AND (“peri-operation”) AND “day surgery” OR “short stay surgery” OR “ambulatory surg*.” We searched BMJ Best Practice, RNAO, NICE, NGC, SIGN, Association of Day Surgery (IAAS), China Alliance for Cooperation in Day Surgery, Cochrane, Joanna Briggs Institute (JBI), Medline, Embase, and CBM. The search time limit was from the establishment of the database to September 2021. The inclusion criteria of the literature were elderly patients (60 years old) who were treated in the day operation ward, and the content of the study was related to perioperative management. The study was designed for clinical decision-making, clinical practice guidelines, expert consensus, evidence summary, and systematic evaluation and was published in Chinese and English. The exclusion standard was that the full text could not be obtained, and the data of the research content report was incomplete.

#### Evidence evaluation

A total of 11 articles were included in this project, including 2 guidelines (13, 14), 4 expert consensus (15–18), and 5 evidence summary (19–23). The quality of the two guidelines was evaluated using the Appraisal of Guidelines for Research and Evaluation (AGREE) (24).

The quality evaluation criteria of four expert consensus were used to evaluate the authenticity of the literature according to the evaluation tools of JBI evidence-based healthcare center expert opinions and professional consensus articles (25). Five pieces of evidence summarized the literature on which the evidence was based, and the corresponding evaluation criteria were selected according to the type of literature for quality evaluation. All the literature studies were scored by three researchers who had received evidence-based training according to the contents of the corresponding literature quality evaluation items, and the controversial items were discussed by the third-party researchers and finally reached an agreement. A total of 26 pieces of evidence of perioperative management of elderly patients undergoing day surgery were extracted.

### Setup of an evidence evaluation team to determine the best evidence and develop audit indicators

The evidence evaluation team was composed of a head nurse in the day operation ward, 3 full-time nurses in the day operation ward, 2 general surgeons, and 1 nursing graduate student, all of whom have bachelor’s degree or above and had more than 2 years of working experience in the day operation ward. Based on the attributes of evidence FAME, the feasibility, suitability, clinical significance, and validity of 26 pieces of evidence were evaluated. The evidence level of the original guide or evidence summary was retained, and their sources were marked. When the included evidence comes from multiple articles, one with a higher level of evidence was selected. Finally, 7 pieces of best evidence were included. According to the principle of merging the same evidence and splitting the different evidence, 11 pieces of evidence for examination had been formulated, as shown in Table 1.

**Table 1:** The audit indicators for the included evidence.

Content of evidence	Source and grade of evidence	Audit indicator	Audit method
1. Confirm the appointment date and time and preoperative intake to the patient by phone before operation, and screen for any diseases that may delay the operation.	JBI, level Ι	1. The appointment nurse in the day operation ward completed the pre-admission screening of elderly patients 1 day in advance to confirm whether they could be admitted to the hospital as scheduled.	On-site verification + nursing record sheet confirmation
		2. The appointment nurse in the day operation ward prepared the health education before operation on the phone.	On-site verification
2. Patients are encouraged to participate in decision-making and to provide preoperative and postoperative information, and distractions such as music, television or magazines should be provided in the waiting area to reduce preoperative anxiety.	JBI, level Ι	3. TV publicity was set up in the waiting area for admission, and the content of perioperative health education for daytime surgery would be broadcast.	On-site verification
		4. Elderly patients were accompanied by their family members and they participated in perioperative care.	On-site verification
3. Health education for day surgery should include effective, repeatable and understandable instructions, guidance and follow-up.	Clinical practice guide, level Ι	5. Nurses in the day operation ward provide patients with perioperative treatment and nursing health education list for corresponding diseases.	On-site verification
		6. Clear follow-up time before discharge and matters needing attention after operation for patients	On-site verification
4. The perioperative waiting time can be reduced by improving information management and coordination among medical teams.	JBI, level ΙΙΙ	7. The waiting time of elderly patients at each stage after admission.	Review the electronic medical record system
		8. The use of electronic admission booking system for doctors	Review the electronic medical record system
5. Assess the risk of postoperative nausea and vomiting and related preventations and treatments.	Clinical practice guide, level Ι	9. To evaluate the incidence of postoperative nausea and vomiting in elderly patients	On-site verification
6. Clear, repeatable and easy-to-operate discharge evaluation criteria should be established for day surgery to ensure patient safety.	Clinical practice guide, level Ι	10. Evaluation of discharge preparation of elderly patients before discharge	On-site verification + questionnaire
7. After discharge from hospital, patients undergoing day surgery need to be followed up to understand their recovery status, to find out postoperative adverse events in time and to guide their management.	Clinical practice guide, level Ι	11. The follow-up nurse in the day operation ward completed the telephone follow-up within 1 week after discharge.	Nursing record sheet confirmation

### Current situation review

We established an evidence-based core group, including a professor engaged in nursing management and evidence-based practice methodology; two nursing backbones who were engaged in day surgery and trained in evidence-based methodology were responsible for team training, project implementation, evidence implementation process control, and data management; and three full-time day surgery nurses with bachelor’s degree were responsible for team coordination and communication, data collection, and follow-up maintenance. Two full-time nurses for day surgery were responsible for the application of evidence.

We collected data on the current nursing situation from September 2021 to December 2021. (1) On-site verification: As shown in Table 1, the review indicators 1–6, 9, and 10 were all judged by the members of the evidence-based core group on the spot of nurses and elderly patients in the day operation ward and truthfully recorded in the project data summary table according to the situation. (2) Questionnaire: As shown in Table 1, index 10 was investigated by the full-time nurses in the designated day operation ward using the Chinese version of the discharge readiness scale (26). Cronbach’s α of the internal consistency of the whole scale is 0.89 with good reliability and validity. (3) Review of the electronic medical record system: As shown in Table 1, review indicators 7 and 8 were extracted from the electronic medical record system, including waiting time for admission, operation, anesthesia start time, operation time, and time out of the operating room. (4) Nursing record sheet confirmation: As shown in Table 1, indicators 1 and 11 were reviewed, and the data collection and follow-up content forms were developed by the members of the evidence-based core group, and full-time nurses in the day operation ward were designated to collect information, including whether they could be admitted to the hospital as scheduled, preoperative fasting and intestinal preparation confirmation, and follow-up after discharge.

### Introduction of evidence

#### Analysis of obstacle factors

The main obstacles discussed and identified by the team included the following points: (1) There were differences in the process of admission preparation among different departments; (2) There was a lack of perioperative health education and materials; (3) The compliance of elderly patients with preoperative preparation was insufficient; (4) Doctor’s acceptance of the electronic day operation booking system was not high; (5) The turnover rate of daytime operation was high, and doctor’s order was not issued in time, which affected the progress of operation, discharge, and patient experience; (6) Medical staff did not have enough understanding of the best evidence-based practice in the perioperative period of day surgery; (7) Accurate evaluation of daytime surgery, there were deficiencies in the selection of appropriate anesthetic methods; and (8) The number of follow-up of patients undergoing daytime operation was large.

#### Implement change

We introduced the best evidence to update the perioperative management process of elderly patients undergoing day surgery. The members of the evidence core group organized the management staff of the medical department, the doctors of the relevant clinical departments, and the nurses in the day operation ward for on-the-spot discussion to introduce the best evidence into the existing perioperative management process of elderly patients undergoing day surgery. The updated contents included unified coordination of preoperative preparation for daytime surgery before admission, evaluation of outpatient anesthesia, second confirmation of admission time, time of issuance of doctor’s orders, form, content, and time of health education, health education materials for different operations, evaluation and observation of postoperative complications, and postoperative follow-up. The updated perioperative management of elderly patients in the day operation ward was based on the best evidence content and defined the executor, execution time, and implementation standard in the whole process of day operation for elderly patients, which was operable.

In addition, we strengthened effective communication between doctors and nurses and introduced a daytime surgery information management system. We set up a WeChat working group for daytime surgery doctors and nurses. The doctor could book the operation date and bed at the same time in the outpatient clinic or complete the appointment after the consultation. Full-time nurses for daytime surgery confirmed the bed reservation information in the information management system and contacted the patients over the phone at least 1 day in advance to confirm the admission time of the patients and carry out preoperative preparation of health education. After the elderly patients were admitted to the hospital, the responsible nurse took the initiative to remind the bed doctor to issue surgical orders, nursing orders, and drug orders in time. The responsible doctor followed up the operation process in the operating room in time and contacted the ward nurse to prepare for the operation. After the operation, we correctly evaluated the patient’s postoperative condition, gave timely feedback to the doctor, and prepared for discharge. We organized and summarized the daily operation turnover every 2 weeks, communicated about the hindering factors, and improved the perioperative management process of elderly patients undergoing day surgery.

We also carried out perioperative management training for elderly patients undergoing day surgery. The perioperative management training of elderly patients undergoing day surgery was carried out through professional study and shift transfer, and the nurses were assessed. The contents included the confirmation of patient appointment information in the daytime operation information management system, the unification of health education content, updating the contents of preoperative preparation and the confirmation of preoperative evaluation results, postoperative nursing and discharge preparation evaluation, and other related knowledge.

At the same time, we have established a perioperative health education and discharge follow-up system for elderly patients undergoing day surgery. The content of perioperative health education for elderly patients undergoing day surgery consists of three parts. According to the best evidence, the appointment nurse and the responsible nurse completed the confirmation of the patient’s operation date and prepared the content of health education before admission to ensure that the operation was carried out normally. Intensive health education was carried out during the operation stage after admission. According to different anesthetic methods, surgical sites, surgical contents of health education, material distribution, and picture and text publicity, including TV rolling broadcast, corridor publicity wall posters, and video health education. After the completion of the operation, the patients were given postoperative health education, including observation of complications, time of intake, and type and usage of drugs. The elderly patients were followed up by the follow-up nurses in the ward within 1 week after discharge, including the incidence of complications, drug use compliance, pathological report review, and outpatient follow-up time.

### Effect evaluation

#### Study population

In this evidence application, the day operation ward of a tertiary hospital in Shanghai was selected, and the subjects were the elderly patients and all the day surgery department nurses. The inclusion criteria for elderly patients were as follows: age ≥ 60 years old; the patients underwent day surgical treatment in our hospital; and the patients agreed to participate in this study.

A total of 10 nurses in the day operation ward were enrolled. The inclusion criteria were as follows: the nurses had the nurse qualification and registered nurse certificate; full-time nurse in the day operation ward, who was familiar with and knew most of the day operation types and nursing care; and the nurses had obtained the informed consent and agreed to participate in this study. The baseline review stage before the application of evidence was from September 2021 to December 2021 and the review stage after the application of evidence was from September 2022 to December 2022.

#### Evaluation index

Practitioner level: Ten nurses in the day operation ward were investigated before and after evidence-based practice with self-made examination studies. The knowledge part included the process of perioperative management of day surgery for the elderly, assessment tools, health education, and postoperative follow-up, with a full score of 100. In addition, we used the Chinese version of the evidence-based practice questionnaire (EBPQ) on knowledge, belief, and practice to investigate clinical nurses (27). It had been reported that EBPQ’s Cronbach’s α was 0.94 and content validity was 0.83 with good reliability and validity (28). Process level: the implementation rate of each examination index of perioperative management of elderly patients undergoing day surgery. The data collection method included on-the-spot verification method, questionnaire method, consulting electronic medical record system, and form confirmation. Patient level: the change in waiting time in the perioperative period of elderly patients.

### Statistical method

SPSS 21.0 statistical software was used for data analysis. The measurement data were expressed by mean and standard deviation using an independent sample *t*-test for group comparison. The counting data were expressed by the number of cases and percentage and compared by chi-square test. In this study, *p* < 0.05 was considered statistically significant.

## Results

This evidence-based practice included 10 full-time nurses in the day operation ward. All the above personnel were trained in evidence-based practice. The level of knowledge, belief, and practice of nurses on perioperative management of elderly patients undergoing day surgery before and after evidence-based practice is shown in Table 2.

**Table 2:** The knowledge, belief, and practice of nurses before and after evidence-based practice.

	Items	Before evidence application	After evidence application	*p*
		Score	Score	
1. Perioperative knowledge mastery	Admission process	100	100	
	Evaluation tool	100	100	
	Examination items	100	100	
	Preoperative education	100	100	
	Postoperative education	95	100	
	Postoperative follow-up	95	100	
2. Evidence-based practical knowledge		54.55 ± 11.73	65 ± 5.75	0.016
3. Evidence-based practice attitude		20.88 ± 4.76	30.21 ± 3.71	0.022
4. Evidence-based practice behavior		24.81 ± 5.72	29.20 ± 2.76	0.008

Before evidence-based practice, the implementation rates of indicators 8 and 10 were 0, and the implementation rates of indicators 1, 3, 5, and 11 were all less than 30%. After evidence-based practice, the implementation rates of all indicators were significantly improved (*p* < 0.05). At the same time, the implementation rate of indicators 6 and 9 reached 100%, as shown in Table 3.

**Table 3:** Comparison of perioperative management audit indicators of elderly day surgery before and after evidence-based practice (%).

Audit indicator	Before evidence application (*n* = 353)	After evidence application (*n* = 348)	*p*
1. The appointment nurse in the day operation ward completed the pre-admission screening of elderly patients 1 day in advance to confirm whether they could be admitted to the hospital as scheduled.	20.68	60.63	<0.01
2. The appointment nurse in the day operation ward prepared the health education before operation on the phone.	0	60.63	<0.001
3. TV publicity was set up in the waiting area for admission, and the content of perioperative health education for daytime surgery would be broadcast.	27.76	43.10	<0.001
4. Elderly patients were accompanied by their family members and they participated in perioperative care.	45.61	86.21	<0.001
5. Nurses in the day operation ward provide patients with perioperative treatment and nursing health education list for corresponding diseases.	14.16	43.10	<0.006
6. Clear follow-up time before discharge and matters needing attention after operation for patients.	97.73	100	
7. The waiting time of elderly patients at each stage after admission.	/	/	/
8. The use of electronic admission booking system for doctors.	0	30.3	<0.001
9. To evaluate the incidence of postoperative nausea and vomiting in elderly patients.	98.02	100	
10. Evaluation of discharge preparation of elderly patients before discharge.	0	57.47	<0.001
11. The follow-up nurse in the day operation ward completed the telephone follow-up within 1 week after discharge.	28.33	43.10	<0.001

Before the evidence-based practice, there were 353 elderly patients, including 234 men and 119 women, and after the evidence-based practice, there were 348 elderly patients, including 247 men and 101 females. There was no significant difference in baseline before and after evidence-based practice (*p* > 0.05), as shown in Table 4. After evidence-based practice, the length of waiting for admission and waiting for operation and returning to the ward to discharge in elderly patients were shorter than those before practice, and the difference was statistically significant (all *p* < 0.05).

**Table 4:** Comparison of perioperative process time before and after the application of evidence.

Variables		Before evidence application		After evidence application		*p*
		Cases	Percentage/mean ± standard deviation	Cases	Percentage/mean ± standard deviation	
Gender	Male	234	66.3	247	79.98	0.103
	Female	119	33.7	101	29.02	
Age			71.65 ± 5.751		70.13 ± 6.721	0.095
Source of operation	Department of urology surgery	206	58.4	213	61.21	0.067
	Department of general surgery	46	13	29	8.33	
	Gastroenterology	23	6.5	16	4.6	
	Department of pain	53	15	30	8.62	
	Department of thoracic surgery	5	1.4	10	2.87	
	Department of eye, ear, ophthalmology & otorhinolaryngology	6	1.7	8	2.30	
	Department of gynecology	1	0.3	23	6.61	
	Department of internal medicine	13	1.4	19	5.46	
Waiting time for admission (h)			101.76 ± 45.63		89.76 ± 40.62	0.002
Length of examination after admission (h)			1.98 ± 2.20		2.98 ± 2.71	0.053
Waiting time for surgery (h)			15.05 ± 10.21		11.05 ± 13.21	0.025
Length of time to receive patients in operating room (h)			0.44 ± 0.17		0.31 ± 0.11	0.114
The time between the arrival of the patient in the operating room and the beginning of anesthesia (h)			0.87 ± 0.57		0.72 ± 0.63	0.206
Length of time from the patient’s return to the ward to discharge after operation (h)			12.27 ± 14.35		11.57 ± 15.27	0.041

## Discussion

Under the premise of emphasizing the coexistence of efficiency and patient safety, daytime surgery can continuously improve the delay of perioperative management, shorten the operation waiting time of elderly patients, and improve the sense of medical experience of patients (29). The sense of experience of patients has a direct impact on their satisfaction and recommendation of hospital services (30). In this study, through evidence-based practice, the perioperative process of day surgery and medical satisfaction for elderly patients are further improved. During the outpatient visit, clinicians often evaluate the actual condition of elderly patients and judge whether it is suitable for daytime surgery. In this process, the time and channels for elderly patients and their families to receive information related to daytime surgery are insufficient, which can easily lead to many feelings of uncertainty and conflict about disease treatment, hospitalization environment, and medical process after admission. According to the evidence content of perioperative management of elderly patients undergoing day surgery, it combines preoperative health education and second confirmation of operation time of patients moving forward, prompting the admission time of patients in advance, improving the preoperative examination, estimating the length of stay in hospital, and suggesting reliable family members to accompany (31, 32). The results of this study show that after evidence-based practice, the implementation of indicators 1, 2, and 4 has been significantly improved. At the same time, the video of perioperative health education was played by rolling in the special waiting area of the daytime operation ward, and the individual perioperative health education was provided by responsible nurses; the result shows that the implementation of indicators 3 and 5 has improved in the review.

The perioperative management of day surgery for the elderly is the result of multi-disciplinary cooperation and multi-sector cooperation on the same public platform (33, 34). Under the policy guidance of the medical administration department, we jointly present the perioperative management mode and content of evidence-based day surgery. Through the introduction of evidence-based evidence, this study promotes the mastery and application of the latest evidence for perioperative management of day surgery in the elderly patients. Doctors and nurses gradually use the “daytime operation management system” to integrate electronic appointment information of patients and confirm the time of admission, the type of disease, the type of operation, and whether the preoperative anesthetic evaluation has been completed. At the management level, the latest best evidence content is strictly practiced in clinical practice, and the homogeneity of platform management is improved, which ensures the quality of day operation management for elderly patients and improves the homogeneity and standardization of day surgical service quality.

Day surgery has been paid more and more attention by researchers in the field of medical and healthcare year by year. The particularity of the elderly population is increasingly prominent in the day surgery medical group, which not only meets the needs of patients but also meets the requirements of the construction of a general hospital day operation platform, so there is an urgent need for the development and application of intelligent management platform (35–37). In order to further optimize the allocation of medical resources, daytime surgery management from extensive to fine transformation improves the level of medical services and advocates the creation of intelligent information on big data platforms (38, 39). Based on the background of the development of deep aging, the evidence of perioperative management in geriatric day operation ward is also the basic framework starting point for the development and construction of an intelligent management platform, in order to make patients get more convenient and effective medical experience and promote the development of high-quality daytime surgery.

Day operation is a type of operation mode that helps the patient to get out of the hospital in a short time. This model can not only reduce the total cost of patients but also improve the utilization rate and work efficiency of medical resources, and it is an efficient and convenient health service model. Although day surgery has been highly concerned and recognized by the medical community, and various medical institutions have made great efforts to carry out day surgery under the call of national policy in China, there are still some problems. It has been recognized by the vast majority of patients, but it is facing many challenges in developing countries, especially the limited coverage of medical insurance and the lack of follow-up services (40, 41). The mode of day operation ward is in the stage of development, and the speed of development is different in different areas (42). The nursing management of daytime surgery is quite different from that of the general ward, which needs to be adjusted according to the characteristics of daytime. This study has summarized the current findings of the literature, in order to provide a reference for our country to continuously improve the nursing management of daytime surgery, maximize the rational use and allocation of medical resources, deepen the concept of high-quality nursing service, and improve the satisfaction of the society, government, patients, and medical staff, to contribute to the reform of the medical and health system.

## Conclusion

In conclusion, the best evidence of perioperative management of elderly day surgery patients was introduced into clinical practice in this study, and the standardized content of perioperative management was established by implementing the perioperative management process of day surgery. With relevant training for medical staff, promotion of daytime operation management system, and improvement of awareness and compliance of perioperative management evidence of medical staff for elderly patients, the nursing quality of daytime surgery has been improved greatly. However, due to the multi-team cooperation in this study, there is still insufficient use of electronic assessment tools and insufficient discharge readiness evaluation, which need to be solved through evidence-based practice in the later stage. In addition, this study is based on the clinical scenario, and the conclusions are limited; it is suggested that the validity of the evidence should be further verified by multicenter studies with a larger sample size in future investigations.

## Data availability statement

The original contributions presented in the study are included in the article/supplementary material, further inquiries can be directed to the corresponding authors.

## Ethics statement

This study had obtained the approval from the ethical committee of our hospital with approval number: 2021K010. The studies were conducted in accordance with the local legislation and institutional requirements. The participants provided their written informed consent to participate in this study.

## Author contributions

MW: Investigation, Methodology, Writing – original draft. LX: Investigation, Writing – review & editing. LZ: Investigation, Writing – original draft, Writing – review & editing. YX: Investigation, Writing – original draft. YC: Investigation, Writing – original draft. XZ: Writing – original draft. LC: Investigation, Writing – original draft.
